# Optical sensing and control of T cell signaling pathways

**DOI:** 10.3389/fphys.2023.1321996

**Published:** 2024-01-10

**Authors:** Hae Nim Lee, Seung Eun Lee, Kyung-Soo Inn, Jihye Seong

**Affiliations:** ^1^ Brain Science Institute, Korea Institute of Science and Technoloy, Seoul, Republic of Korea; ^2^ Department of Converging Science and Technology, Kyung Hee University, Seoul, Republic of Korea; ^3^ Department of Pharmacology, Seoul National University College of Medicine, Seoul, Republic of Korea; ^4^ Wide River Institute of Immunology, Seoul National University, Hongcheon, Republic of Korea

**Keywords:** T cell receptor, CAR-T, genetically encoded fluorescent biosensor, FRET, optogenetics

## Abstract

T cells regulate adaptive immune responses through complex signaling pathways mediated by T cell receptor (TCR). The functional domains of the TCR are combined with specific antibodies for the development of chimeric antigen receptor (CAR) T cell therapy. In this review, we first overview current understanding on the T cell signaling pathways as well as traditional methods that have been widely used for the T cell study. These methods, however, are still limited to investigating dynamic molecular events with spatiotemporal resolutions. Therefore, genetically encoded biosensors and optogenetic tools have been developed to study dynamic T cell signaling pathways in live cells. We review these cutting-edge technologies that revealed dynamic and complex molecular mechanisms at each stage of T cell signaling pathways. They have been primarily applied to the study of dynamic molecular events in TCR signaling, and they will further aid in understanding the mechanisms of CAR activation and function. Therefore, genetically encoded biosensors and optogenetic tools offer powerful tools for enhancing our understanding of signaling mechanisms in T cells and CAR-T cells.

## 1 Introduction

T cells play a pivotal role in adaptive immune response by recognizing a wide variety of antigens via T cell receptor (TCR) signaling pathways (Gaud et al., 2018; Xu et al., 2020; Shah et al., 2021). In addition, chimeric antigen receptor (CAR) T cell therapy has been developed by combining the functional domains of T cell receptor (TCR) with the antibodies that specifically recognize cancer antigens (Irving and Weiss, 1991; Porter et al., 2011; Pan et al., 2022). TCR complex is primarily responsible for these functions, thus it is crucial to understand how the activation of TCR and the downstream signaling pathways are regulated with spatiotemporal resolutions (Choudhuri and van der Merwe, 2007).

To study the molecular mechanisms of TCR activation and the related signaling pathways, traditional methods have been employed such as Western blot, flow cytometry and immunostaining. They have provided valuable insight into the TCR signaling pathways, however these methods require the fixation of the cells, making it difficult to investigate the dynamic molecular mechanisms of the TCR signaling pathways in live cells (Im et al., 2019). In contrast, recent advances in live-cell imaging techniques and genetically encoded fluorescent biosensors have allowed the real-time monitoring of molecular events of TCR signaling pathways with spatiotemporal resolutions (Kim et al., 2021). In addition, optogenetic tools based on photosensitive proteins can control dynamic molecular events by light, providing the further understanding of the molecular mechanisms of complex signaling pathways (Seong and Lin, 2021).

This review aims to introduce fluorescent biosensors (Section 4) and optogenetic tools (Section 5) for investigating dynamic T cell signaling pathways in live cells. Section 3 outlines previous experimental methods employed in studying these pathways, with considerations on their limitations, potentially addressed by employing fluorescent biosensors and optogenetic tools. In addition, Sections 1 and 2 have been incorporated to provide readers with essential background on T cell signaling pathways initiated by TCR and CAR, enabling them to track which segments of the pathways are explored using fluorescent biosensors and optogenetic tools. Finally, the review concludes by proposing future directions to advance these technologies for achieving a deeper comprehension of T cell signaling pathways.

## 2 The signaling pathways of T-cell receptor (TCR) complex

**FIGURE 1:**
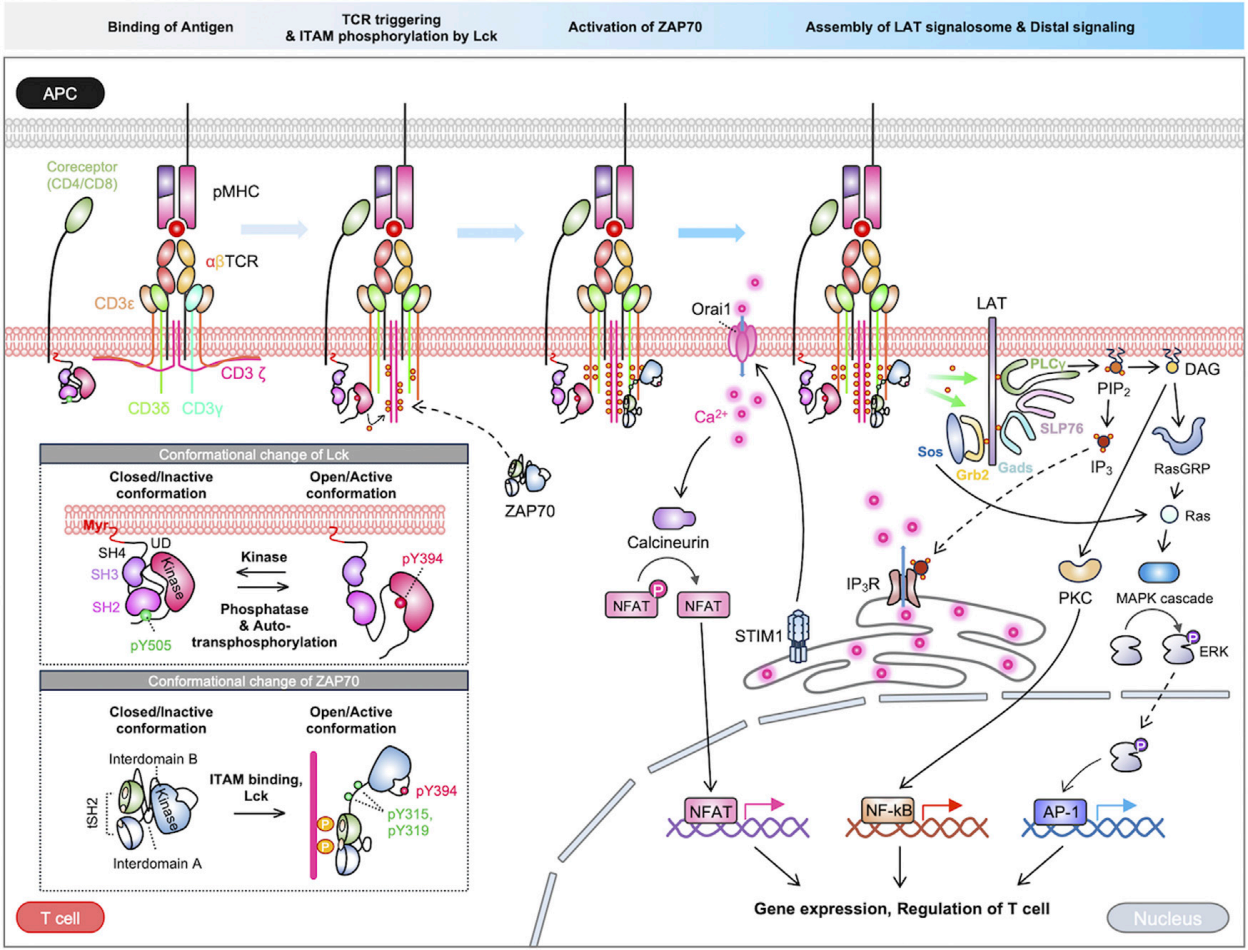
Overview of TCR signaling pathways. The TCR signaling is initiated by the binding of an antigen peptide-MHC complex. The process of signal transduction from the extracellular to intracellular domain is a topic of ongoing debate, with multiple proposed models. Following TCR triggering, the kinase Lck phosphorylates the ITAM motif within the TCR complex. ZAP70 is subsequently recruited to the phosphorylated ITAMs, where it undergoes partial activation, then ZAP70 is further enhanced by Lck. The activated ZAP70 phosphorylates LAT, which recruits various signaling molecules. The LAT signalosomes initiate cascades of downstream pathways, ultimately activating crucial transcription factors like NFAT, NF-κB, and AP-1. These pathways collectively regulate various T-cell functions.

T cell receptors recognize a variety of antigens and initiate signaling pathways for adaptive immune responses (Huse, 2009). The TCR complex is a sophisticated assembly of integral membrane proteins incorporating highly variable α and β chains, which noncovalently associated with CD3 signaling subunits γ, δ, ε, and ζ (Choudhuri and van der Merwe, 2007; Xu et al., 2020; Gangopadhyay et al., 2022). TCR signaling pathways are initiated by the interaction between the α/β chains of the TCR complex and the antigen peptides presented on major histocompatibility complex (MHC) in antigen-presenting cells (APCs). This interaction triggers the activation of TCR, subsequently resulting in the phosphorylation of CD3 signaling domains called immunoreceptor tyrosine-based activation motifs (ITAMs). Consequently, the downstream signaling cascades including Lck (lymphocyte-specific protein tyrosine kinase), ZAP70 (zeta chain-associated protein kinase 70), LAT (linker for the activation of T cells), lead to the propagation of TCR signaling pathways (Courtney et al., 2018). In this section, we describe the sequences of TCR activation and downstream signaling pathways (Figure 1).

### 2.1 Recognition of antigen peptide-MHC complex

The first step of the TCR activation is the recognition of antigen peptide-MHC complexes presented by APCs. From antigen processing process, APCs cleave long antigens into short antigen peptides and these short peptides bound to MHC (pMHC) are displayed on the APC surface (Vyas et al., 2008; Pishesha et al., 2022). TCRs recognize the pMHC via the complementarity determining regions (CDRs) in the variable domains of the TCR αβ chains (Rossjohn et al., 2015). The variable domains are highly diversified through random genetic recombination of variable (V) and joining (J) gene segments, and with the exclusive presence of diversity (D) gene in the β chains, making the TCRs to distinguish and recognize a wide repertoire of antigen peptides (Huang et al., 2012; Szeto et al., 2020; Shevyrev et al., 2022). The binding structure of TCR-pMHC is further stabilized by co-receptors CD4 and CD8 of T cells, forming the ternary complex of TCR-pMHC-co-receptors (Morch et al., 2020; Rushdi et al., 2022).

### 2.2 TCR triggering process

Upon the recognition of antigen peptide, the TCR initiates the transduction of signals across the plasma membrane, a process called TCR triggering (Dushek, 2011; van der Merwe and Dushek, 2011). While the precise mechanisms remain incompletely understood, several models of the TCR triggering process have been proposed, for example, serial engagement, kinetic proofreading, segregation, and conformation change (Courtney et al., 2018). It is important to note that these models are not mutually exclusive; rather, they coexist and complement each other contributing to a comprehensive understanding of the intricate orchestration of TCR signaling events.

The serial engagement model proposes that rapid re-binding of a single peptide-MHC to multiple TCRs can initiate TCR signaling. (Valitutti et al., 1995; Valitutti, 2012). In this model, a pMHC binds to a TCR causing a degree of signaling, then the dissociated pMHC repeatedly binds to other TCRs, thereby triggering the TCR signaling. This model provides insight into how T cells respond sensitively to even a single agonist pMHC. The *kinetic proofreading model* postulates that the persistent binding of pMHC to TCRs for adequate duration is required for the initiation of TCR signaling (Aleksic et al., 2010; Courtney et al., 2018; Pettmann et al., 2021; Britain et al., 2022). For example, a pMHC-TCR complex with high affinity can persist for adequate time to be scanned by the co-receptor, thus resulting in the initiation of TCR signaling. Conversely, a complex with low affinity may dissociate before being scanned by the co-receptor. The *kinetic segregation model* proposes that the spatial rearrangement of signaling molecules on the plasma membrane triggers the TCR signaling. In this model, the exclusion of CD45 from the APC contact zone containing TCR-pMHC complexes leads to an increase activity in Lck. This facilitates the phosphorylation of the TCR complex, thereby initiating the process of TCR signaling (Anton van der Merwe et al., 2000; Davis and van der Merwe, 2006; Malissen and Bongrand, 2015).

The *conformational change model* proposes that TCRs bound to pMHC change their conformation triggering intracellular signaling cascades. Different hypotheses have been suggested regarding the mechanisms of the conformational changes of TCR. First, the mechanical force generated from the pMHC pulling on the αβ chains of TCRs may be propagated through the intracellular CD3 subunits, as structural evidences have demonstrated the rigid and cohesive interaction with TCRαβ and CD3 subunits (Kim et al., 2012). Optical tweezers further revealed that lateral force of 50 pN induces a conformational change of the TCR complex and potentiates the signaling pathways (Feng et al., 2017; Stephens et al., 2022). Another mechanism for the conformational change of TCR is the release of ionic interaction between the basic residue-rich sequence (BRS) in the CD3 subunits of the TCR complex and the negatively charged phospholipids in the inner leaflet of the plasma membrane. The engagement of TCRs to pMHC triggers the influx of Ca^2+^, which can neutralize the negative charge of the anionic phospholipids. Subsequently, the ionic interaction between the phospholipids and the CD3 domains of the TCR complex is released thus the cytosolic functional domains of CD3 are dissociated from plasma membrane to initiate the TCR signaling pathways (Shi et al., 2013).

In these TCR triggering models, it has been suggested that the TCRs are further clustered for their signaling pathways (Varma et al., 2006; Huang et al., 2013; Taylor et al., 2017). For example, Huang et al. found that a single pMHC induces the slow formation of TCR clusters, and the TCR clustering makes more favorable environment for the pMHC to serially engage with other TCRs (Huang et al., 2013). It was also shown that the ligands with longer bound-time can promote the TCR clustering, and the receptor clustering is crucial for signaling outputs in the *kinetic proofreading* model (Taylor et al., 2017). Furthermore, Minguet et al. have suggested that conformational change of TCR alone is not sufficient to fully activate TCR signaling pathways unless combined with the TCR clustering (Minguet et al., 2007). These findings demonstrate that TCR clustering is an important step for the TCR signaling pathways.

### 2.3 Immunological synapse formation

The engagement of TCR with the pMHC complex and the subsequent TCR clustering initiate the reorganization of membrane receptors and signaling molecules, leading to the dynamic formation of immunological synapse (Monks et al., 1998; Grakoui et al., 1999; Huppa and Davis, 2003; Dustin, 2014). This process is finely orchestrated by regulatory proteins and membrane microdomains such as lipid rafts (Jury et al., 2007; Wu et al., 2016). The immunological synapse exhibits a distinct structure with specific molecular compositions (Monks et al., 1998; Huppa and Davis, 2003). The central supramolecular activation cluster (cSMAC) positioned at the core of the immunological synapse contains the clustered TCRs, associated signaling molecules (e.g., CD3 and ZAP-70), and co-receptors (e.g., CD4 or CD8). Surrounding the cSMAC, the peripheral SMAC (pSMAC) comprises adhesion molecules such as LFA-1 and ICAM-1, facilitating stable cell-cell interactions. The distal SMAC (dSMAC), situated at the periphery, includes inhibitory phosphatase CD45. This spatial organization of signaling molecules within immunological synapse is crucial for initiating downstream pathways (Dustin and Cooper, 2000; Thauland and Parker, 2010; Garcia and Ismail, 2020), and thus immunological synapse can serve as a platform for T cell signaling pathways.

### 2.4 The phosphorylation of ITAM motifs by Lck

The next step of TCR signaling pathways is the phosphorylation of ITAMs in the CD3 cytoplasmic domains by Lck kinase (Irving et al., 1993; Clements et al., 1999; Love and Hayes, 2010). It has been suggested that Lck exists in two distinct states: a bound state Lck which is associated with the coreceptors CD4 and CD8 (Kim et al., 2003), and an unbound state Lck anchored to the cell membrane via myristoylation and palmitoylation (Ehrlich et al., 2002; Zimmermann et al., 2010). Because co-receptors engage both pMHC and Lck, the bound state Lck can be targeted to TCR to phosphorylate the ITAMs in the CD3 cytoplasmic domains (Li et al., 2004; Bommhardt et al., 2019; Morch et al., 2020). Recently, it has been suggested that the initial phosphorylation of ITAM motifs can be facilitated by the unbound state Lck (Casas et al., 2014). In fact, Lck may be directly recruited to the TCR complex as shown by multiple interactions between Lck and the CD3 domains of the TCR complex. For example, the BRS region in the CD3εζ can directly interact with acidic residues in the unique domain (UD) of Lck (Li et al., 2017). In addition, multiple anchorage points have been identified between the receptor kinase (RK) motif of CD3ε and the SH3 domain of Lck (Hartl et al., 2020). Therefore, Lck is able to phosphorylate the ITAMs in CD3, initiating downstream signaling pathways of TCR.

### 2.5 Activation of ZAP70

The phosphorylated ITAM motifs in the CD3 domains of the TCR complex provides the binding site for the tandem SH2 (tSH2) domains of ZAP70, a 70 kDa tyrosine kinase. It comprises tSH2 domain, a C-terminal catalytic domain, and two interdomains; interdomain A, which connects the tSH2 domain, and interdomain B, bridging the tSH2 domain and the catalytic domain (Hatada et al., 1995). In the resting state, ZAP70 resides predominantly in the cytoplasm as an autoinhibited form. In contrast, the recruitment of ZAP70 to the phosphorylated ITAMs at the plasma membrane triggers the partial alleviation of its autoinhibitory conformation (Yan et al., 2013). Lck further phosphorylates the interdomain B linker (Y315 and Y319 residues) and kinase domain (Y493) of ZAP70, stabilizing the active conformation of ZAP70 (Wang et al., 2010; Yan et al., 2013; Courtney et al., 2018). The fully activated ZAP70 directs its kinase activity towards two essential scaffold proteins; the transmembrane linker for the activation of T cells (LAT) and the cytoplasmic SH2 domain–containing leukocyte protein of 76 kDa (SLP76) (Bubeck Wardenburg et al., 1996; Zhang et al., 1998).

### 2.6 Assembly of LAT signalosome and distal signaling

LAT is a major adaptor protein phosphorylated by ZAP70 kinase. It comprises a short extracellular domain, followed by a transmembrane segment and an elongated cytoplasmic domain. The cytoplasmic domain of LAT harbors nine tyrosine residues. The phosphorylated tyrosine residues can serve as the docking sites for a variety of signaling molecules, for example, Grb2 (growth factor receptor-bound protein 2)/Sos (son of sevenless) and PLCγ1 (phosphor-lipase C gamma1), therefore resulting in the assembly of LAT signalosomes (Paz et al., 2001; Samelson, 2002).

SLP76 is another crucial adaptor protein phosphorylated by ZAP70. SLP76 is characterized by three domains, a N-terminus region featuring three tyrosine motifs, a proline-rich region, and a C-terminal SH2 domain (Balagopalan et al., 2010). The phosphorylated tyrosine residues in the N-terminus recruit Vav, a guanine-nucleotide-exchange factor (GEF), an adaptor protein Nck, and a member of the Tec family kinase Itk. Additionally, the proline-rich domain of SLP76 interact with Gads and PLCγ1, culminating in the formation of LAT-Gads-SLP76-PLCγ1 complex (Koretzky et al., 2006).

An array of proteins recruited to LAT initiates a cascading sequence of downstream pathways, thus enhancing signal diversification and amplification of the TCR signaling pathways (Balagopalan et al., 2015). For example, activated PLCγ1 hydrolyzes phosphatidylinositol 4,5 biphosphate (PIP_2_) into diacylglycerol (DAG) and inositol 1,4,5-triphosphate (IP_3_) (Katan, 1998; Chuck et al., 2010). DAG can activate PKC and RasGRP1 (Ras Guanyl Releasing Protein 1), ultimately leading to the activation of transcription factors, NF-kB (nuclear factor kappa-light-chain-enhancer of activated B cells) and AP-1 (activator protein 1), respectively (Shah et al., 2021). On the other hand, IP_3_ binds to Ca^2+^-permeable ion channel receptors (IP_3_R) on the endoplasmic reticulum (ER), inducing the release of Ca^2+^ from the ER to the cytoplasm (Jayaraman et al., 1996). The elevated intracellular Ca^2+^ activates calcineurin, a protein phosphatase that dephosphorylates the nuclear factor of activated T cells (NFAT), leading to its translocation into the nucleus (Trebak and Kinet, 2019). Thus, Ca^2+^ is an essential component for the T cell effector functions (Joseph et al., 2014; Trebak and Kinet, 2019). Collectively, the TCR signaling pathways regulate proliferation, migration, cytokine production, and effector functions through activation of diverse transcription factors (Figure 1) (Ashouri et al., 2022).

## 3 Chimeric antigen receptor (CAR)-mediated signaling pathways

Chimeric antigen receptor (CAR) T cell therapy is an innovative cancer treatment which reprograms the patient’s T cells with CAR to specifically identify and destroy cancer cells on their own (Frederickson, 2015). The architecture of CAR comprises a single-chain variable fragment (scFv), a hinge region, a transmembrane domain, and the intracellular signaling domains including the ITAMs derived from CD3ζ and costimulatory domains (Sterner and Sterner, 2021). CAR-T cell therapy has revolutionized the field of cancer immunotherapy with promising outcomes in clinical trials for hematological cancers (Pan et al., 2022), however, the activation mechanisms of CAR-T signaling pathways remain incompletely understood.

In the intracellular part, CAR contains the signaling domains derived from TCR. Thus, it is anticipated that the CAR signaling pathways are generally similar to the ones of TCR, such as the ITAM phosphorylation and the recruitment of ZAP kinase (Gudipati et al., 2020; Wu et al., 2020; Wang et al., 2022). However, Dong et al. reported a potentially novel mechanism of CAR signaling independent of LAT, which is distinct from the LAT-dependent TCR signaling pathways. They found that CARs directly engage the LAT binding partners for example, Grb2, Gads, and PLCγ1, thereby leading to downstream signaling events such as Ras activation, actin remodeling, and Ca^2+^ influx (Dong et al., 2020).

In the extracellular part, CAR contains the scFv instead of the TCR αβ chains, thus the initial activation mechanism of CAR would be distinct from the TCR activation mechanism. For example, the scFv in the CAR exhibit high affinity for antigen, characterized by the nanomolar range of dissociation constant (KD) (Kammertoens and Blankenstein, 2013; Harris et al., 2018; Mao et al., 2022), while TCR exhibits much lower affinity for antigen peptide (1–100 μM of KD). In contrast, the sensitivity of CAR is lower than the one of TCR. It has been suggested that the activation of TCR can be initiated by as few as one pMHC whereas CAR typically requires at least hundreds of antigens to elicit an effective response (Watanabe et al., 2015; Wu et al., 2020; Yun et al., 2023). These distinct features may result in different activation mechanism of CAR from TCR.

CAR incorporates one or two costimulatory domains derived from costimulatory proteins of T cells such as CD28 and 4-1BB (Honikel and Olejniczak, 2022). Proteomic analysis has revealed that the activation of CAR containing CD28/CD3ζ or 4-1BB/CD3ζ led to nearly identical changes in phosphorylation patterns (Salter et al., 2018). The distinction lies in the markedly enhanced kinetics of signaling, as shown in the pre-clinical results that the CD28-containing CARs demonstrate faster in antitumor activity while also exhibiting exhaustion when compared to the 4-1BB-containing CARs (Zhao et al., 2015; Salter et al., 2018; Cappell and Kochenderfer, 2021). Recent study has elucidated that the relatively slow kinetics of the 4-1BB-containing CAR may be due to the specific association of the THEMIS (thymocyte selection associated)-SHP1 (Src homology region 2 domain-containing phosphatase-1) complex with the 4-1BB domain, which leads to the dephosphorylation of CAR-CD3ζ ultimately resulting in the attenuation of the CAR signaling (Sun et al., 2020).

## 4 Traditional methods for studying TCR or CAR-T signaling pathway

TCR was discovered in the early 1980s and the first-generation CAR was developed in the early 1990s (Eshhar et al., 1993). Extensive efforts have been dedicated to unravel the intricate T cell signaling pathways. Investigating the T cell signaling pathways has relied on conventional methodologies, primarily encompassing Western blotting, flow cytometry, and immunofluorescence staining. This section provides an overview of conventional methodologies used to investigate TCR/CAR signaling. Furthermore, this section address the limitations of these methods which could potentially be addressed by introduction of fluorescent biosensors and optogenetic tools, the topics that will be elaborated in the upcoming Section 5.

### 4.1 Western blotting

Western blotting allows for the analysis of protein expression and post-translational modifications (Mahmood and Yang, 2012; Pillai-Kastoori et al., 2020; Begum et al., 2022). To investigate the role of CD3ζ in TCR signaling, Irving and Weiss conducted an experiment where they designed a chimeric protein by combining the extracellular and transmembrane domains of CD8 with the cytoplasmic domain of CD3ζ. Through Western blot analysis, they observed that the phosphorylation of CD3ζ could initiate signal transduction, emphasizing the pivotal role of CD3ζ as a signal transducer (Irving and Weiss, 1991). Importantly, the research suggests that even when substituting the αβ chains of the TCR with alternative antigen recognition motifs, such as the single-chain variable fragment (scFv) of a CAR, the T cell signaling pathways can persist. Extending these observations, Chan et al. verified that the phosphorylated CD3ζ of both the TCR and the CD8/ζ chimera associates with ZAP70 (Chan et al., 1991). Paz et al. demonstrated that ZAP70 directly phosphorylates LAT, with the phosphorylated sites in LAT serving as docking sites for various proteins (Paz et al., 2001). These findings, exemplified through Western blotting techniques, have significantly advanced the comprehension of TCR and CAR signaling pathways.

Western blotting, however, requires lysis of the cells to obtain protein samples, therefore it does not provide spatiotemporal information about target proteins in live cells. In addition, Western blot results should be interpreted carefully since samples may also contain unwanted cells.

### 4.2 Flow cytometry

Flow cytometry stands as a crucial single-cell technique, offering high-throughput assessments of light scattering and fluorescent signals from individual cells (McKinnon, 2018). Unlike Western blotting, which provides average measurements for the entire cell population (He and Fox, 1996), flow cytometry excels in revealing heterogeneity in protein amounts. Gating techniques are employed in flow cytometry to isolate and analyze specific cell populations of interest (Adan et al., 2017; McKinnon, 2018; Staats et al., 2019). The activation of TCR/CAR, measurable through increased activation markers such as CD69 (McKinnon, 2018), is extensively explored using flow cytometry (Bray et al., 2018; Sun et al., 2020; Cassioli et al., 2021; Lo et al., 2023).

Despite its versatility in immunophenotyping and protein quantification, flow cytometry has limitations. It heavily relies on fluorescence analysis, often requiring compensation for signal interference (Roederer, 2002; Szaloki and Goda, 2015; Adan et al., 2017; Staats et al., 2019). Furthermore, flow cytometry is constrained in its ability to analyze protein dynamics in a single cell level with a high spatiotemporal resolution.

### 4.3 Immunofluorescence staining

Immunofluorescence staining enables the precise detection of the localization of target proteins or post-translational modifications through specific antibodies (Im et al., 2019). This method offers insights into the distribution of proteins within fixed cells or tissues. Subsequent examination of the stained samples is typically conducted through fluorescence microscopy, providing the spatial information of target proteins or post-translational modifications with high precision. Using this method, Monk et al. identified distinctive protein localization within the immunological synapse, defining it by two concentric rings of molecules known as cSMAC and pSMAC (Monks et al., 1998). Furthermore, Lee et al. revealed that early phosphorylation events of Lck and ZAP-70 precede the establishment of a mature immunological synapse, utilizing anti-pLck or anti-pZAP-70 antibodies (Lee et al., 2002). This emphasizes that T cell engagement and activation initiate before the complete formation of immunological synapses (Lee et al., 2002; Saito and Yokosuka, 2006). In a recent investigation, the comparative analysis of immunological synapse structures between TCR and CAR within the same T cell has yielded valuable insights. The immunological synapse induced by CAR exhibited a distinctive and disorganized multifocal pattern of Lck arrangement, along with a small actin ring and lacked distinct LFA-1 distribution. This unique immune synapse configuration correlated with accelerated tumor target cell killing and efficient detachment from dying tumor cells by CAR-T cells (Davenport et al., 2018).

However, the utilization of this method necessitates the fixation of cells to prevent decay and autolysis while preserving antigenicity. Consequently, this fixation step poses a limitation, impeding the study of dynamic protein interactions in living cells. (Im et al., 2019).

## 5 Genetically-encoded biosensors for monitoring the activity of TCR and CAR

Genetically encoded biosensors with fluorescent proteins (FPs) have emerged as powerful tools for monitoring dynamic biological events within living cells. These biosensors utilize physicochemical properties of FPs, such as fluorescence spectra, maturation time, pH sensitivity, photoconversion, and fluorescence resonance energy transfer (FRET) (Kim et al., 2021). For example, FRET is a photophysical phenomenon driven by energy transfer between closely positioned (<10 nm) donor and acceptor FPs of overlapping spectral profiles. Thus, proteins of interests can be fused to donor and acceptor, and their interaction can be measured by FRET signal in live cells.

Innovative fluorescent biosensors have been developed and employed to visualize intricate molecular mechanisms of T cell functions with high spatiotemporal resolutions. For example, they enabled the real-time monitoring of the conformational changes of TCR during its activation (Sasmal et al., 2020), spatiotemporal enzymatic activities of downstream molecules (Randriamampita et al., 2008; Paster et al., 2009; Stirnweiss et al., 2013; Cadra et al., 2015; Philipsen et al., 2017), and the trans-localization of transcription factors in live cells (Regot et al., 2014). On the other hand, the field of CAR-T cell therapy has primarily focused on enhancing their therapeutic effects, but the molecular mechanisms underlying their activation and regulation remain poorly understood. It is crucial to unveil these mechanisms for the development of safe and more effective CAR-T cell therapy, thus fluorescent biosensors will be valuable tools to investigate the mechanisms of CAR signaling pathways. In this section, we review the genetically encoded biosensors for the monitoring of each step of the TCR signaling pathways. Additionally, we explore the potential applications of these biosensors for the CAR research field.

### 5.1 Antigen recognition

**FIGURE 2:**
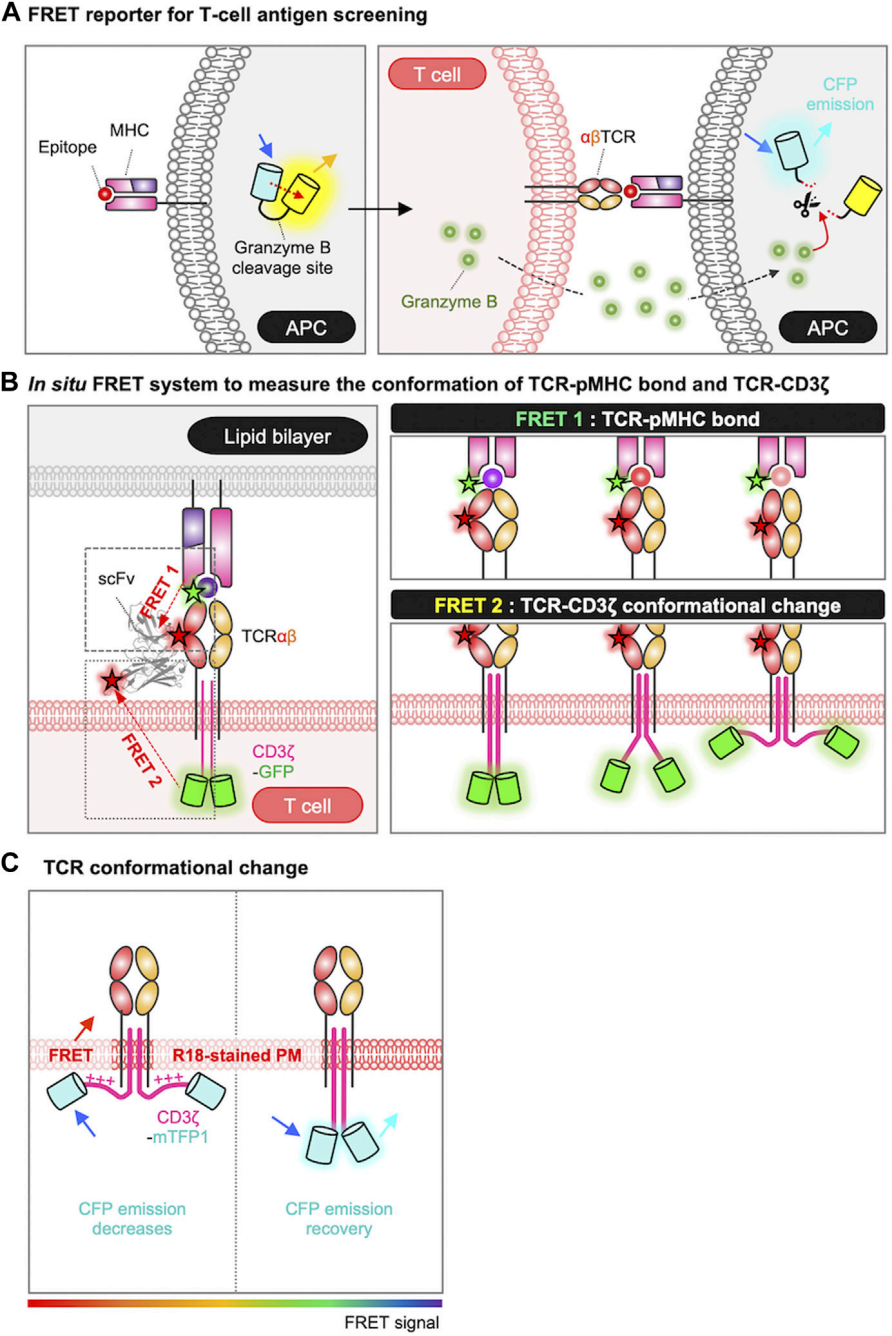
Genetically encoded biosensors for detecting antigen recognition and TCR triggering process. **(A)** Schematic representation of FRET-based screening system for the epitope of TCR. In the absence of granzyme B, the FRET biosensor emits a FRET signal between CFP and YFP. When the epitope is recognized by the TCR, granzyme B released from the T cell enters the APC and cleaves the substrate site of the FRET biosensor. The loss of the FRET signal, combined with the rescue of the CFP signal, is detected by FACS and enables the isolation of cells undergoing T-cell targeting (Sharma et al., 2019). **(B)** Measurement of TCR conformational dynamics by FRET. The conformational dynamics of the TCR-pMHC bond are measured using FRET 1, and the conformational change of TCR-CD3ζ can be measured using FRET 2. For FRET 1, TCR is labeled with Cy5 via scFv J1, and the C-terminus of the peptide is labeled with Cy3. For FRET 2, TCR is labeled with Alexa Fluor 568 via scFv J3, and the C-terminus of CD3ζ is tagged with GFP. The pMHC molecules are anchored to a lipid bilayer or a PEG-Ni^2+^ glass surface (Sasmal et al., 2020). **(C)** FRET design to detect conformational changes in CD3ɛ/ζ cytoplasmic domains. FRET occurs when CD3ɛ/ζ chains, tagged with mTFP, associate with the plasma membrane, labeled with the R18 dye (Xu et al., 2008; Zhang et al., 2011; Li et al., 2017). The rainbow color bar indicates the FRET levels, where warm and cool colors indicate high and low FRET levels, respectively.

As discussed in Section 2, the first step in TCR activation is the recognition of the antigen peptide by MHC molecules on APCs. For the identification of novel epitope from peptide library, Sharma et al. developed a FRET-based epitope screening system (Figure 2A) (Sharma et al., 2019). In this system, epitope-encoding minigene libraries were introduced in APCs which also expressing a FRET-based granzyme biosensor. The granzyme biosensor is composed of a FRET pair, CFP (cyan fluorescent protein) and YFP (yellow fluorescent protein), linked by a specific substrate sequence for granzyme B. CFP and YFP are proximal displaying strong FRET in the default state, in contrast, the FRET decreases when active granzyme B cleaves the substrate in the FRET biosensor. When the presented epitope is recognized by TCR, the activated T cells release granzyme B which then cleaves the substrate sequence of the FRET biosensor resulting in the decrease of FRET signal. These changes in FRET can be detected by flow cytometry, thus this FRET-based high-throughput screening of T cell antigen peptides from the libraries on a scale of 10^6^ enabled the identification of novel epitope sequences for TCR.

To investigate the interaction between TCR and pMHC at the T cell membrane *in situ*, researchers utilized a method based on single-molecule FRET (smFRET) between an acceptor fluorophore attached to the TCR (scFv-Cy5) and a donor fluorophore labeled on the pMHC (pMHC-Cy3) (Figure 2B) (Huppa et al., 2010; Sasmal et al., 2020). By plotting the histogram of FRET efficiencies for individual smFRET trajectories and fitting it with a Gaussian function, they derived the intermolecular distances between TCR and pMHC: 44 ± 9 Å for a strong agonist (K5), 54 ± 11 Å for a agonist (MCC), and 66 ± 18 Å for a weak agonist (102S). Thus, the binding strength between TCR and pMHC can be evaluated by smFRET measurement. In addition to the TCR-pMHC binding property (FRET 1), the release of CD3ζ from the plasma membrane during TCR triggering process can be also measured by *in situ* FRET system (FRET 2) (Figure 2B) (Sasmal et al., 2020). The FRET 2 is further explained in Section 5.2.

CAR directly recognizes target antigen through its scFv domain (Guedan et al., 2019; Rafiq et al., 2020), and the binding affinity between the CAR-scFv and target antigen are measured by *in vitro* methods such as ELISA and surface plasmon resonance (SPR). For the screening of CAR-scFv which strongly binds to target antigens expressed on the surface of cancer cells, a luciferase-based method called Malibu-Glo assay has been developed (Natarajan et al., 2020). In this assay, target antigen-expressing cells are incubated with various scFv-luciferase fusion proteins, unbound proteins are removed, then the bound scFv-luciferase can be measured by luminescence. Therefore, Malibu-Glo assay allows efficient cell-based screening of scFv candidates for desired binding capacity. This method is still limited to accurately mimic the cell-to-cell interactions between CAR-T and cancer cells, thus advanced techniques to evaluate their binding property will facilitate the development of effective CAR-T therapy.

### 5.2 TCR triggering process

After TCR engagement, the TCR triggering process occurs which is believed to initiate intracellular signaling cascades. Several working models include serial engagement, kinetic proofreading, segregation, and conformation changes (Li et al., 2017; Courtney et al., 2018; Xu et al., 2020). Previous studies provided *in vitro* evidence of the TCR triggering process using X-ray crystallography, nuclear magnetic resonance (NMR), and cryogenic electron microscopy (CryoEM) (Mariuzza et al., 2020) or indirect measurements with synthetic lipids (Aivazian and Stern, 2000).

To visualize the conformational change of TCR during the triggering process in live cells, the FRET technique was applied by labeling of the cell membrane with octadecyl rhodamine B chloride (R18) dye and tagging of CD3ɛ/ζ cytoplasmic domains with mTFP (monomeric teal fluorescent protein) (Figure 2C) (Xu et al., 2008; Zhang et al., 2011; Li et al., 2017). The researchers successfully measured the FRET between mTFP and R18, confirming the association between the CD3 chains and the inner plasma membrane at the resting state of T cells. They also demonstrated that this association of CD3 chains to the plasma membrane occurs through electrostatic interactions between the BRS domains in the CD3 chains and negatively charged phospholipids at the inner plasma membrane (Xu et al., 2008; Zhang et al., 2011; Li et al., 2017). Furthermore, the FRET signal was significantly reduced in the contact region with CD3-coated beads, suggesting the dissociation of CD3 chains from the inner plasma membrane upon the TCR engagement (Zhang et al., 2011).

In addition, *in situ* FRET system has further allowed the real-time monitoring of the conformational change of TCR-CD3ζ (Figure 2B, FRET 2) (Sasmal et al., 2020). In this system, TCRβ was labeled with scFv-Alexa Fluor 568 and the C-terminus of the CD3ζ chain was tagged with GFP. Thus, the intramolecular distance between TCR and pMHC was measured by FRET2 and the one between TCR and CD3ζ was detected by FRET1 (Figure 2B). The data demonstrated that the tighter TCR–pMHC bond induced by a strong agonist can lead to the significant dissociation of CD3ζ from the inner plasma membrane. Consequently, this leads to the exposure and phosphorylation of the ITAM regions in the CD3ζ domain, and ultimately initiates the TCR signaling pathways.

### 5.3 TCR clustering and immunological synapse formation

It is suggested that the engaged TCR-pMHC complex tends to form clusters. To study the TCR clustering, we can utilize homologous FRET (homo-FRET), a phenomenon occurring between proteins with identical fluorophores (Bader et al., 2009; Bader et al., 2011), because the protein clustering can be measured by the changes in fluorescence anisotropy due to homo-FRET (Weidtkamp-Peters et al., 2022). Using this technique, Rocheleau et al. quantified the clustering levels of MHC-I on the endoplasmic reticulum and the cell surface (Rocheleau et al., 2003). In another investigation, researchers developed a sensor named CD3ζ-CliF (clustering reported by intermolecular FRET), capable of assessing the TCR clustering through intermolecular FRET between neighboring FRET pairs (Ma et al., 2017). Using CD3ζ-CliF, they tracked that TCR triggering can increase the number of the TCR-CD3ζ clusters, and observed the movement of the clusters within immunological synapses.

Emerging evidence suggests that the size and density of TCR clusters are strongly correlated with the initiation of TCR signaling (Yokosuka et al., 2005; Pageon et al., 2016; Goyette et al., 2019), underscoring the importance of visualization of TCR clusters in the study of TCR signaling. However, accurate detection or quantification of the size/density of TCR clusters has been challenging due to the limitation in resolution of conventional microscope. Recent advances in super-resolution imaging techniques, such as such as stimulated emission depletion (STED) microscopy and light-sheet microscopy (LSM), allows the more profound understanding in the T cell biology by visualizing TCR at the single-molecule level (Luo et al., 2020; Rochussen et al., 2023). For example, these techniques have revealed that the previously observed TCR nanoclusters in resting T cells (Lillemeier et al., 2010; Pageon et al., 2016) may be artifacts arising from the image reconstruction process or the coating material used to adhere cells onto a glass surface (Ponjavic et al., 2018; Rossboth et al., 2018).

In addition to TCR clustering, the subsequent spatial rearrangement of other receptors, adhesion molecules and signaling molecules at the plasma membrane triggers the formation of immunological synapse (Dustin, 2014), a crucial platform for TCR signaling pathways. Immaging the immunological synapse between cells has been challenging due to the requirement of for *en face* reconstruction at the contact site in the xy plane with limited spatial resolution. To address this, artificial substrates such as antibody-coated slides and supported lipid bilayers (SLB) are commonly used, however these methods may not fully replicate the natural immunological synapse between cells. To overcome these limitations, a combination of optical tweezers and confocal microscopy has been employed to visualize immunological synapse in living cell conjugates with high speed and resolution (Oddos et al., 2008). This method achieves an *en face* view of the synapse by optically trapping conjugated cells and manipulating the orientation of the cell conjugates in the imaging plane of a laser scanning confocal microscope.

### 5.4 The phosphorylation of ITAM motifs by Lck

#### 5.4.1 Lck activation

The ITAM regions in the CD3ζ are phosphorylated by active Lck kinase (Rossy et al., 2012). Lck, a member of Src family kinases, is composed of an N-terminal SH4 domain for myristoylation and palmitoylation, unique domain (UD), SH3 and SH2 domains, a proline-rich region, a catalytic kinase domain, and a C-terminal tail. Lck can anchor to the plasma membrane through the SH4 domain, facilitating its diffusion within the inner leaflet of the plasma membrane (Yurchak and Sefton, 1995). The enzymatic activity of Lck is tightly regulated by the phosphorylation and dephosphorylation of two key tyrosines, Tyr394 in the kinase domain and Tyr505 in the C-terminal tail (Yamaguchi and Hendrickson, 1996). Lck exists as a closed conformation in the inactive state by stable interaction between pTyr505 and the SH2 domain (Xu et al., 1999; Boggon and Eck, 2004). During TCR activation, this pTyr505 can be dephosphorylated by CD45 and thus released from the SH2 domain, allowing the primed open conformation of Lck (Saunders and Johnson, 2010). Subsequently, the exposed Tyr394 in the activation loop of the kinase domain can be autophosphorylated, resulting in the full activation of Lck (Hui and Vale, 2014).

**FIGURE 3:**
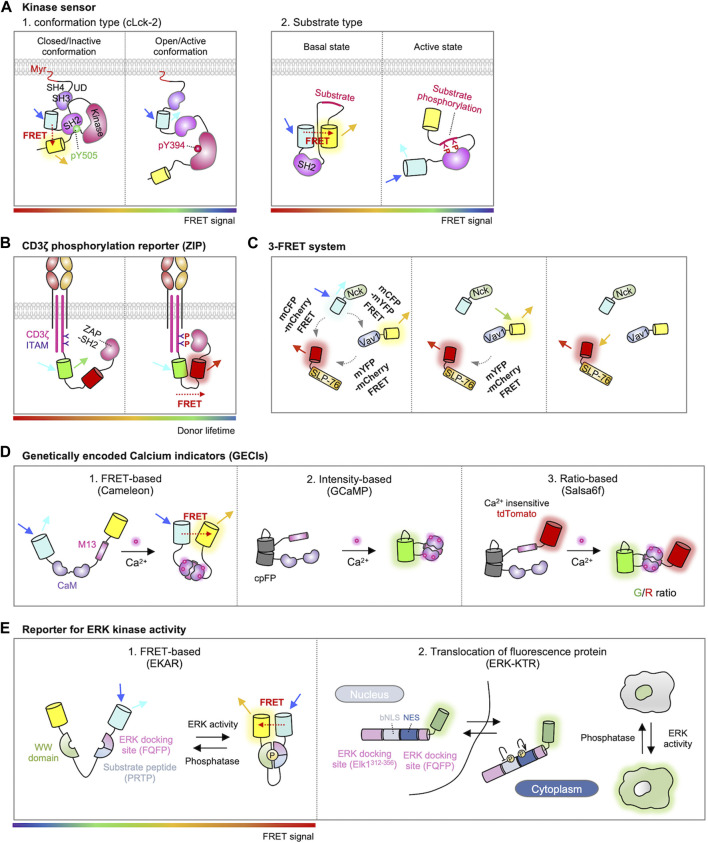
Genetically encoded biosensors for detecting T cell signaling. **(A)** Two types of FRET-based kinase sensors. The first type, conformational biosensors, monitors conformational changes within a kinase upon activation (left) (Paster et al., 2009). An example is cLck-2, a probe for Lck, which includes CFP inserted between the SH3 and SH2 domains and YFP located behind the kinase domain of Lck. This design enables the detection of conformational alterations in Lck. When Lck is in a closed/inactive state, the probe displays a high FRET signal; conversely, in the open/active conformation, it exhibits a low FRET signal. The second type, substrate biosensor, employs a kinase-inducible molecular switch (right) (Wan et al., 2019). This mechanism links endogenous kinase activity to the FRET efficiency of the biosensor. When the substrate within the sensor is phosphorylated by the target kinase, the SH2 domain binds to it, initiating a rearrangement of the sensor and consequently modifying the FRET signal. The rainbow color bar indicates the FRET levels, where warm and cool colors indicate high and low FRET levels, respectively. **(B)** Schematic design of the ZIP reporter. The ZIP reporter consists of eGFP, mCherry, and ZAP70-SH2 domains. When the ITAM domain is phosphorylated by T cell activation, ZAP70-SH2 binds to the tyrosine-phosphorylated ITAM, inducing FRET between eGFP and mCherry, resulting in a decrease in eGFP fluorescence lifetime (Yudushkin and Vale, 2010). The rainbow color bar indicates the FRET levels, where warm and cool colors indicate high and low FRET levels, respectively. **(C)** Schematic representation of the 3-FRET system for studying the dynamics of SLP-76, Nck, and Vav1 multimolecular complex formation. In this system, SLP-76, Nck, and Vav1 are individually tagged with mCherry, mCFP, and mYFP, respectively. The dynamics of their molecular interactions and complex formation are measured by detecting FRET signals between CFP-YFP, CFP-mCherry, and mYFP-mCherry (Pauker et al., 2012). **(D)** Genetically encoded calcium indicators (GECIs). The FERT-based Ca^2+^ sensor is developed by inserting CaM-M13 between donor and acceptor fluorophores such as CFP-YFP or BFP-GFP (Miyawaki et al., 1997). The cpFP based sensor is intensiometric. In this design, a cpFP is inserted between CaM and M13 (Akerboom et al., 2009). The ratio-based sensor closely resembles cpFP-based sensors but includes an additional reference FP, thus measures the Ca^2+^ levels through ratiometric analysis (Dong et al., 2017). **(E)** Representations of ERK kinase activity reporters. In FRET-based ERK kinase sensor, the substrate peptide domain (PRTP) is phosphorylated by ERK, leading to WW domain binding and subsequent conformational change of the sensor. This in turn exhibits a high FRET ratio (left, modified EKAR sensor is shown) (Komatsu et al., 2011). ERK kinase activity can also be quantified by translocation of fluorescence proteins. When ERK is inactive state, the ERK-KTR biosensor is mainly localized in the nucleus. The activated ERK phosphorylates the ERK-KTR biosensor, promoting the translocation of the biosensor outside the nucleus (Right) (Regot et al., 2014). The rainbow color bar indicates the FRET levels, where warm and cool colors indicate high and low FRET levels, respectively.

Based on these conformational changes in the Lck, genetically encoded fluorescent biosensors have been developed for the monitoring of real-time Lck activity during T cell signaling pathways (Paster et al., 2009; Stirnweiss et al., 2013; Philipsen et al., 2017). For example, CLck-Y1 and CLck-Y2 include an acceptor EYFP in the C-terminus and a donor ECFP either in the N-terminal or C-terminal SH3 domain, respectively (Figure 3A) (Paster et al., 2009). In the closed conformation of Lck, strong FRET signals are observed between two FPs, which markedly decreases when Lck adopts the active open conformation. Stirnweiss et al. further performed fluorescence lifetime imaging microscopy (FLIM) experiments using CLck-Y1 biosensor (Stirnweiss et al., 2013), demonstrating that active Lck kinase is already present at rest while the active population of Lck locally increases upon the TCR engagement with pMHC.

Another Lck biosensor, named ZapLck biosensor, consists of an ECFP as the donor, an Src SH2 domain, a tyrosine substrate for Lck kinase (ZAP70 FY), and a YPet as the acceptor (Figure 3A) (Wan et al., 2019). In the basal state, the ZapLck biosensor is expected to exhibit a strong FRET signal between the donor and acceptor. Upon the activation of endogenous Lck kinase, the substrate in the ZapLck biosensor can be phosphorylated by Lck. Then the phosphorylated substrates bind to the intramolecular SH2 domain, separating ECFP and YPet thus decreasing FRET signals. The ZapLck biosensor was used to confirm that a significant population of preactivated Lck exists in Jurkat T cells.

#### 5.4.2 The phosphorylation of ITAM

Active Lck in the contact region of the TCR engagement phosphorylates the tyrosine residues in the ITAMs of CD3ζ chain (Love and Hayes, 2010). As ZAP70 kinase is recruited to the phosphorylated ITAMs, this molecular interaction can be measured by FRET between CD3ζ and ZAP70 labeled with donor and acceptor FPs (Hashimoto-Tane et al., 2010). In addition, Yudushkin et al. developed FRET-based Zeta chain ITAM Phosphorylation (ZIP) reporters (Figure 3B) (Yudushkin and Vale, 2010). ZIP reporters consist of CD3ζ-GFP and ZAP70-SH2 domain tagged with mCherry, either in an intramolecular or intermolecular manner. Upon the TCR activation, the mCherry-tagged ZAP70-SH2 specifically binds to the phosphorylated CD3ζ-GFP, resulting in an increased FRET signal. They further observed with the ZIP reporters the accumulation of phosphorylated CD3ζ in the compartments of endosomal recycling networks (Yudushkin and Vale, 2010), suggesting the sustained TCR signaling at the internalized endosomes (Willinger et al., 2015; Onnis and Baldari, 2019). Using ZIP reporters, another group demonstrated that the internalized TCRs within IRAP (insulin responsive aminopeptidase)-positive endosomes continue to propagate TCR signaling for efficient T-cell responses (Evnouchidou et al., 2020).

### 5.5 Activation of ZAP70

After recruited to the phosphorylated ITAMs, ZAP70 kinase is phosphorylated by Lck to be fully activated (Neumeister et al., 1995; Gangopadhyay et al., 2020), then phosphorylates downstream signaling proteins such as LAT and SLP-76 (Yan et al., 2013). To detect the ZAP70 kinase activity in live cells, Randriamampita et al. developed a FRET biosensor called ROZA (Reporter Of ZAP70 Activity) (Randriamampita et al., 2008). The ROZA reporter contains an N-terminal palmitoylation sequence from Lck, a substrate sequence from LAT, a SH2 domain of Grb2 that can bind to the phosphorylated LAT, and a FRET pair CFP and YFP. Activated ZAP70 kinases phosphorylate the ROZA substrate sequence, which binds to the Grb2-SH2 domain in the biosensor, resulting in a strong FRET between CFP and YFP. The ROZA biosensor revealed that ZAP70 is not only activated at the TCR-APC interface (synapse) but also at the opposite side (anti-synapse), providing important information about spatiotemporal dynamics of ZAP70 activity during TCR signaling pathways. This biosensor was further improved by optimizing the substrate sequence or replacing a FP for FRET (Cadra et al., 2015).

### 5.6 Assembly of LAT signalosome and distal signaling

#### 5.6.1 Assembly of LAT signalosome

ZAP70 phosphorylates LAT which recruits various signaling molecules such as Grb2/Sos and PLCγ1. As discussed above, the FRET-based ZAP biosensor ROZA can monitor the recruitment of Grb2 to the phosphorylated LAT, as well as the activity of ZAP kinase.

ZAP70 also phosphorylates the tyrosine residues of SLP-76. It has been suggested that the phosphorylated SLP-76 recruits Nck and Vav, resulting in a complex of these three molecules (Bubeck Wardenburg et al., 1998; Koretzky et al., 2006). This trimolecular complex is crucial for the cytoskeletal rearrangement during migration, polarity, and proliferation of T cells (Barda-Saad et al., 2010). To investigate the dynamics of this trimolecular complex, Pauker et al. developed a triple-color FRET system in which SLP-76, Nck, and Vav1 were each tagged with mCherry, mCFP, and mYFP, respectively (Figure 3C) (Pauker et al., 2012). Using this 3-FRET system, the dynamics of complex formation and their molecular interactions were assessed by FRET between CFP-YFP, CFP-mCherry, or mYFP-mCherry. Interestingly, the results revealed the formation of Nck-Vav1 dimers without SLP-76 phosphorylation in unstimulated T cells. In addition, in the presence of phosphorylated SLP-76, they observed that Nck binds to SLP-76 and subsequently to Vav1, resulting in the formation of a trimolecular complex.

#### 5.6.2 Distal signaling: Ca^2+^

The LAT signalosome initiates and amplifies the TCR downstream pathways (Horejsi et al., 2004; Courtney et al., 2018). For example, PLCγ1 is recruited to the phosphorylated LAT and initiates the Ca^2+^-related signaling pathways. In addition, SLP-76 binds to and activates Itk, a Tec-family kinase, which then phosphorylates PLCγ1. Upon activation, PLCγ1 generates IP_3_ and DAG, and IP_3_ stimulates the release of Ca^2+^ from ER into the cytoplasm. The increased level of intracellular Ca^2+^ leads to the activation of various downstream signaling pathways, such as calcineurin and a transcription factor NFAT.

Genetically encoded Ca^2+^ indicators (GECIs) have been developed for the monitoring of Ca^2+^ dynamics (Perez Koldenkova and Nagai, 2013; Walia et al., 2018). Cameleon is the first GECI, which is composed of a Ca^2+^ binding protein calmodulin (CaM), a CaM-binding peptide from myosin light-chain kinase (M13), and a FRET pair FPs (Figure 3D) (Miyawaki et al., 1997). The Ca^2+^-bound CaM of the Cameleon interacts with the nearby M13, resulting in increased FRET signals, thus the real-time Ca^2+^ dynamics can be monitored in live cells.

In addition, circular permuted FP (cpFP)-based GECIs include GCaMP series, GECOs, and Camgaroo (Baird et al., 1999; Nakai et al., 2001; Zhao et al., 2011) (Figure 3D). GFP can be circularly permuted as its N- and C-termini are located on the same side. In GCaMP, new N- and C-termini near chromophore are linked to M13 and CaM, thus the interaction between Ca^2+^-bound CaM and M13 can induce the increase of fluorescence (Akerboom et al., 2009). cpFP-based biosensors use single spectrum and their molecular sizes are smaller than the ones based on FRET, and they display high signal-to-noise ratios (Perez Koldenkova and Nagai, 2013). Different expression levels of cpFP-based GECIs can be normalized by adding a reference FP. For example, Salsa6f is GCaMP6f fused with TdTomato (Figure 3D) (Dong et al., 2017). By measuring the ratios of GCaMP6f/TdTomato (G/R), the Ca^2+^ levels can be calculated independent of different expression levels.

#### 5.6.3 Distal signaling: ERK

The TCR signaling pathways activate transcription factors such as NFAT, NF-κB, and AP-1. For example, AP-1 is activated through RasGRP-Ras-ERK pathway (Shah et al., 2021). As discussed above, PLCγ1 is recruited to the LAT signalosome during TCR signaling pathways, producing DAG which further activates RasGRP and Ras (Ebinu et al., 1998; Jun et al., 2013). Activated Ras initiates the mitogen-activated protein kinase (MAPK) signaling cascades, thereby activating the serine-threonine kinases ERK (Kolch, 2005), and ERK kinase activity contributes to activation of a transcription factor AP-1 (Kida et al., 2005; Navarro and Cantrell, 2014).

For detecting ERK activity, Harvey et al. developed a FRET biosensor termed EKAR (Extracellular signal-regulated Kinase Activity Reporter) (Harvey et al., 2008). The EKAR reporter is composed of a FRET pair, EGFP and mRFP1, along with a substrate peptide derived from Cdc25C (Proline-Arginine-Threonine-Proline, PRTP) and the proline-directed WW domain (Figure 3E). To enhance the specificity, an ERK-specific docking site (Phenylalanine-Glutamine-Phenylalanine-Proline, FQFP) was introduced adjacent to the substrate sequence. Activation of ERK prompts the phosphorylation of the substrate sequence in the biosensor, followed by subsequent intramolecular binding between the phosphorylated substrate and the WW domain. This leads to the conformational change of the biosensor, resulting in the FRET signal. The biosensor was further improved by substituting the FRET pair EGFP/mRFP1 with ECFP/YPet or Turquoise-GL/YPet, as well as by optimizing the linker sequences with a longer and more flexible version such as EV linker (Komatsu et al., 2011).

In addition to the FRET-based ERK biosensors, a single-color reporter called ERK-KTR (kinase translocation reports) was innovatively designed by Regot et al. (Figure 3E) (Regot et al., 2014). They found that a suboptimal bipartite nuclear localization signal (bNLS) is negatively regulated by phosphorylation, while the phosphorylation on the nuclear export signal (NES) sequence augments its nucleus export activity. Thus, the ERK-KTR integrates a negatively phospho-regulated NLS with a positively phospho-regulated NES, along with an ERK-specific docking site, a substrate of ERK, and a FP for the visualization. In the default state, the ERK-KTR predominantly localizes within the nuclear compartment, while the phosphorylated ERK-KTR upon ERK activation can translocate to the cytosol. Thus, the activity of ERK can be quantified by measuring the ratio of cytoplasmic to nuclear fluorescent intensity. These sensors are successfully applied to measure ERK activity in T cells (Dong et al., 2021).

## 6 Optogenetic strategies for the fine control of the TCR and CAR signaling with spatiotemporal resolutions

Genetically encoded biosensors enabled the real-time monitoring of dynamic signaling events in live cells with spatiotemporal resolutions, advancing our understanding on the mechanisms of the TCR functions. In addition, natural photosensitive proteins, such as channelrhodopsin 2, light-oxygen-voltage-sensing (LOV) and cryptochrome 2 (CRY2), have been applied for the fine control of molecular events with spatiotemporal manners (Seong and Lin, 2021). Thus, optogenetics with these photosensitive proteins further offers deeper insights into the complex process of TCR and CAR activation and functions. In this section, we introduce optogenetic strategies which have been employed for the study of TCR and CAR functions.

### 6.1 Light-induced multimerization for TCR activation

**FIGURE 4:**
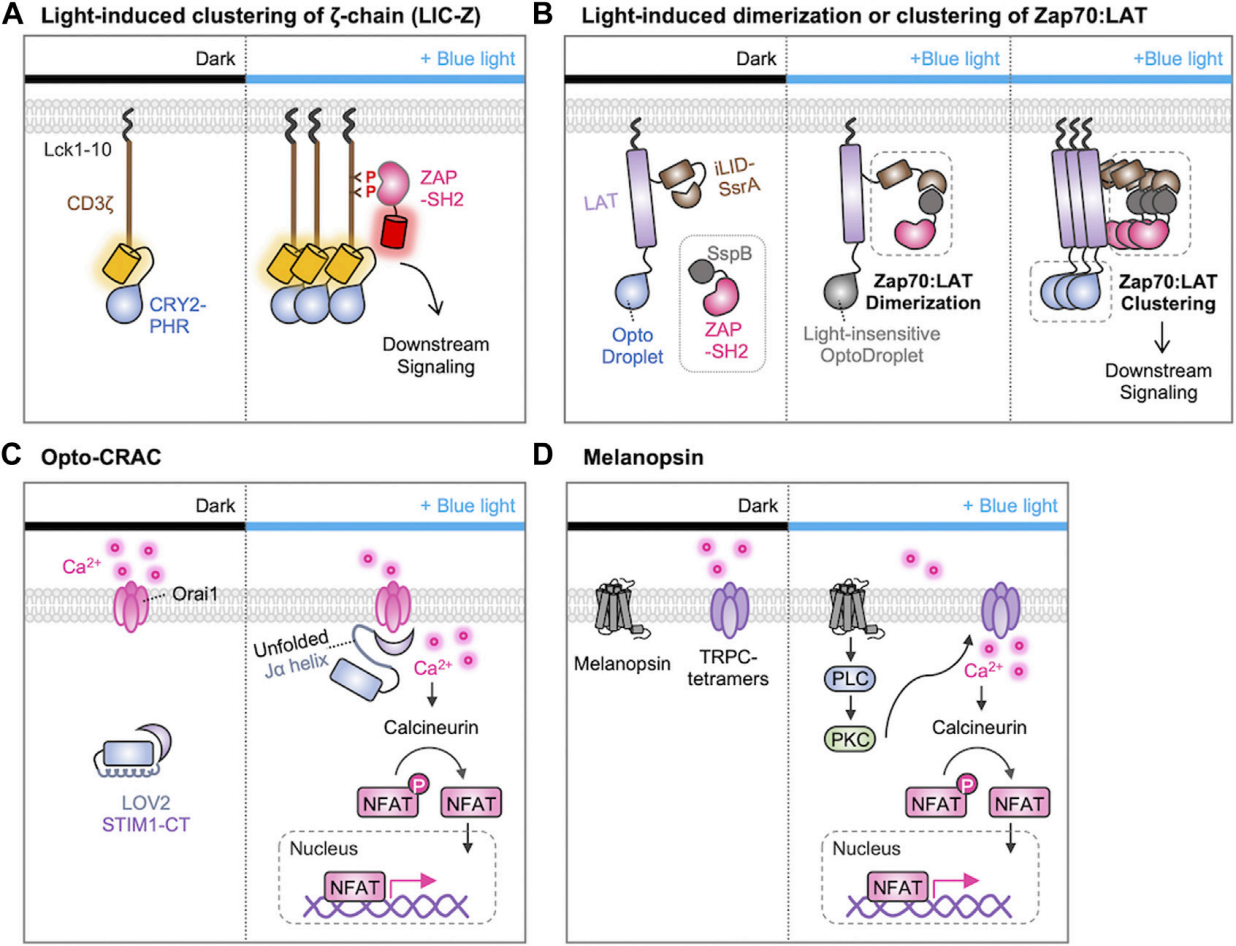
Optogenetic control of T cell activation by protein clustering and Ca^2+^ influx. **(A)** Light-induced clustering of ζ-chain (LIC-Z) consists of a membrane anchor (Lck 1-10 amino acids), the cytosolic domain of CD3ζ, and the light-sensitive CRY2-PHR domain. When exposed to blue light, the LIC-Z clusters and CD3ζ triggering is detected through the translocation of ZAP70-SH2-mCherry to the plasma membrane. This occurs because ZAP70-SH2 specifically binds to phosphorylated ITAMs on LIC-Z. The light-induced clustering was sufficient to initiate various downstream signaling processes (Ma et al., 2020). **(B)** Light-induced dimerization and clustering of ZAP70 and LAT. The light-induced ZAP70-LAT dimerization is prompted by the interaction of LAT-iLID-SsrA and SspB-ZAP70. The light-insensitive OptoDroplet cannot induce their clustering, and no downstream signaling is activated (left). In contrast, OptoDroplet can induce the clustering of LAT-ZAP70, initiating the downstream signaling pathways (right) (Dine et al., 2021). **(C, D)** Optogenetic control of T cell activation through Ca^2+^ signaling with **(C)** Opto-CRAC (He et al., 2015) or **(D)** melanopsin (Zhao et al., 2019). **(C)** In the dark, STIM1-CT remains unexposed by LOV2 domain. Upon blue light illumination, conformation of the C-terminal Jα helix in LOV2 changes to expose the STIM1-CT fragments. This allows them to engage with ORAI1 Ca^2+^ channels, initiating Ca^2+^ influx through the plasma membrane. **(D)** In the absence of light, melanopsin remains inactive. When exposed to blue-light, melanopsin undergoes conformational changes, initiating a cascade of events including activation of Gα_q_, phospholipase C (PLC), and phosphokinase C (PKC). This sequential activation leads to the influx of Ca^2+^ through transient receptor potential channels (TRPCs). Ultimately, Ca^2+^ influx activates the phosphatase calcineurin, dephosphorylating NFAT and initiating nuclear translocation of NFAT.

After the recognition of the antigen peptide-MHC complex, it has been suggested that the TCR clustering at the immunological synapses is crucial for initiating TCR downstream signaling cascades (Cochran et al., 2000; Minguet et al., 2007; Pageon et al., 2016; Taylor et al., 2017). To test this hypothesis, Ma et al. used the light-induced clustering property of a photosensitive protein CRY2 (Ma et al., 2020). In this system, the photosensitive photolyase homology region (PHR) domain of CRY2 was linked to the CD3ζ-chain of the TCR, and they were localized at the plasma membrane via the N-terminal myristoylation sequence (Figure 4A). Upon the illumination of blue light, they observed that the clustering of CD3ζ-chains due to the homo-oligomerization of CRY2-PHR domains, and the CD3ζ clustering induced the TCR downstream cascades such as phosphorylation of ZAP70, PLCγ, ERK, and Ca^2+^ influx. Thus, optogenetic approach demonstrated that the TCR clustering itself is sufficient to initiate TCR signaling.

In addition, a sophisticated optogenetic system was designed to determine if the ZAP recruitment to LAT is sufficient for the initiation of the TCR signaling pathways or if their additional clustering is further required for the T cell activation (Figure 4B) (Dine et al., 2021). Dine et al. used the improved light-induced dimer (iLID)-SspB system to induce the recruitment of ZAP70 to LAT by light. Because LAT-iLID and SspB-ZAP70 can be dimerized by blue light, the authors can finely control the formation of ZAP70-LAT complexes. In addition, they integrated this iLID-SspB system with the CRY2-based optoDroplet system by fusing the CRY2 to the C-terminus of LAT. Therefore, the clustering of ZAP70-LAT complexes can be further induced via CRY2 oligomerization. Importantly, the light-insensitive mutant optoDroplet (D387A) could not induce the clustering of ZAP70-LAT complexes. The results revealed that the formation of ZAP70-LAT complexes is not sufficient, but their further clustering is required for the TCR signaling. Moreover, they uncovered that the light-induced clustering of LAT alone sufficed to trigger a calcium response in T cells, highlighting the importance of molecular clustering in the initiation of TCR signaling pathways.

### 6.2 Light-induced Ca^2+^ modulation for TCR or CAR activation

We previously discussed the importance of Ca^2+^ dynamics in the TCR signaling pathways. Ca^2+^ dynamics is regulated by various ion channels, including Ca^2+^ release-activated Ca^2+^ (CRAC) channels (Shaw and Feske, 2012; Vaeth et al., 2017; Vaeth et al., 2020). It has been suggested that a CRAC channel ORAI1 is activated upon the depletion of Ca^2+^ within the ER. The loss of Ca^2+^ in the ER is detected by stomal interaction molecule 1 (STIM1), which induces the unfolding of its cytoplasmic domain allowing the subsequent interaction with ORAI1. Thus, the STIM1-bound ORAI1 opens the pore of the channel enabling the influx of Ca^2+^ (Lunz et al., 2019).

Optogenetic tools have been designed to finely control Ca^2+^ dynamics and investigate its role in the TCR signaling pathways. For example, He et al. developed Opto-CRAC system to control the ORAI1-STIM interaction, by a photosensitive LOV2 domain fused to the cytoplasmic domain of STIM1 (STIM1-CT) (Figure 4C) (He et al., 2015). In the dark state, the Jα-helix strongly binds to the PAS core in the LOV2, preventing the binding of STIM1 to ORAI1. Upon the blue light, the Jα-helix unfolds and the liberated STIM1 can bind to ORAI1 channels. Thus, Opto-CRAC can control the Ca^2+^ influx by light, which can further induce the Ca^2+^-dependent and NFAT-mediated gene expression in T cells.

Human melanopsin (hOPN4), a member of opsins, is known to mediate the light-inducible intracellular Ca^2+^ mobilization via the PLC/PKC signaling cascade (Peirson and Foster, 2006). In addition, hOPN4 may activate transient receptor potential channels (TRPCs), thereby enhancing Ca^2+^ influx (Peirson and Foster, 2006; Hankins et al., 2008). Zhao et al. demonstrated that optogenetic control of T cell responses can be achieved by the introduction of hOPN4 and the NFAT-mediated cytokine constructs (Figure 4D) (Zhao et al., 2019). In this system, light stimulation can induce the expression of desired cytokines through Ca^2+^ signaling, thus resulting in the enhanced effector T-cell functions for the eradication of solid tumors in a mouse model. Therefore, these optogenetic strategies were successful in delivering Ca^2+^ signals with high spatiotemporal resolutions, inducing the key physiological responses dependent on Ca^2+^/NFAT signaling.

### 6.3 Optogenetic control of expression or activation of CAR

CAR-T cells is designed to specifically target the T cells to the antigen on cancer cells, however they can also bind to normal cells expressing the low levels of target antigens leading to “on-target, off-tumor” cytotoxicity (Sun et al., 2018; Flugel et al., 2023). In addition, side effects such as cytokine release syndrome and neurotoxicity may occur due to the hyperactivation of CAR-T cells (Freyer and Porter, 2020; Siegler and Kenderian, 2020). Therefore, it would be beneficial if the function of CAR-T cells can be precisely controlled both spatially and temporally.

**FIGURE 5:**
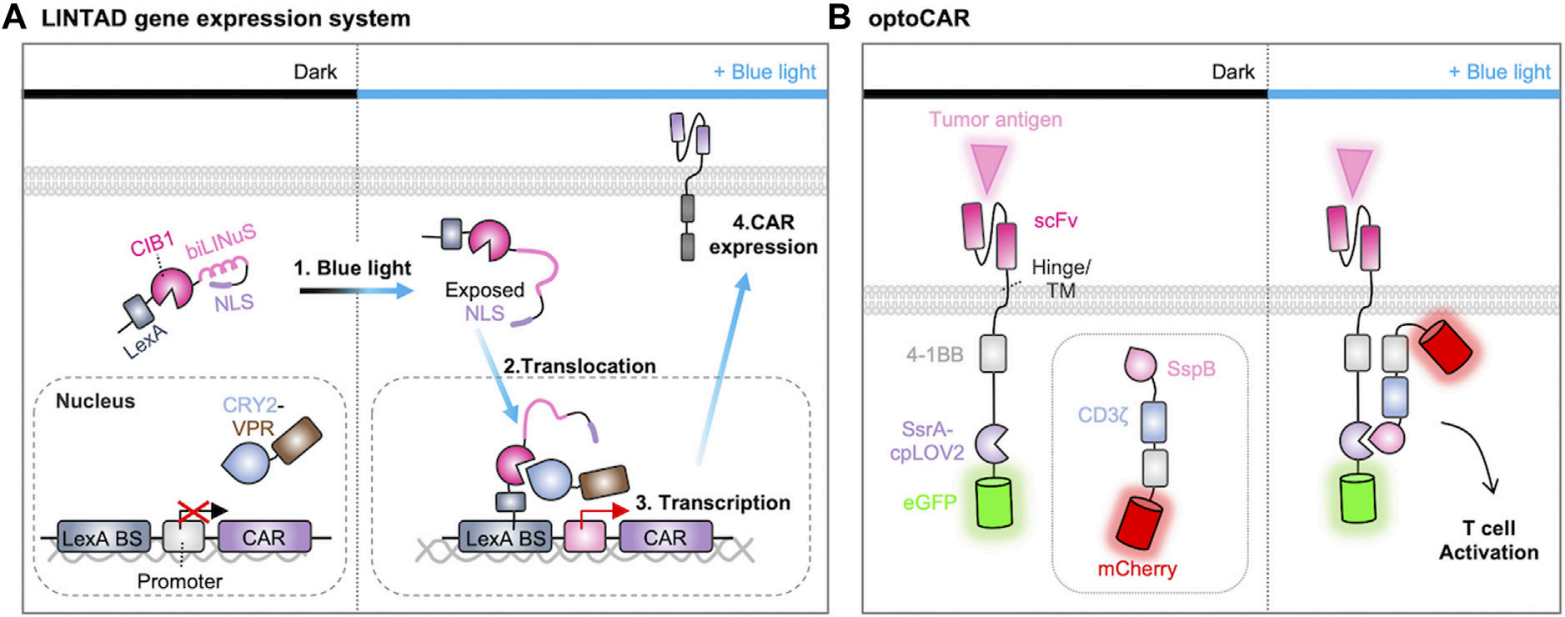
Optogenetic control of CAR expression and activation. **(A)** Light-induced gene transcription and CAR gene expression system. When exposed to blue light, the Jα helix in the LOV2 domain unfolds, revealing the nuclear localization signal (NLS) peptide. This prompts the nuclear translocation of LexA-CIB1. LexA then binds to the LexA-binding sequence on the reporter gene. Blue light also induces the binding of Cryptochrome 2 (CRY2) to CIB1, directing VPR (a transcriptional activator of VP64-p65-RTA) to the minimal promoter of the reporter gene, thus initiating the expression of CAR (Huang et al., 2020). **(B)** Light-induced assembly of optoCAR. The signaling domains 4-1BB and CD3ζ of CAR are expressed as two separate constructs fused to a pair of optical dimerizers (ssrA-cpLOV2 or sspB). Upon exposure to blue light, the optical dimerizer brings the two fragments into close proximity, facilitating their assembly into a functional CAR. This restoration of function enables the T cell to effectively target and eliminate tumor cells (He et al., 2021).

For example, an optogenetic strategy to control the gene expression of CAR construct was successfully designed. Huang et al. developed the system called light-inducible nuclear translocation and dimerization (LINTAD) by integrating the LOV2-based light-inducible nuclear localization signal (biLINuS) and the CRY2-CIB1 (cryptochrome-interacting basic-helix-loop-helix) dimerization system (Figure 5A) (Huang et al., 2020). In the dark state, the LexA-CIB1-biLINuS (LCB) complex is localized in the cytoplasm because its nuclear localization signal (NLS) is masked by LOV2, while the CRY2-VPR (CV) resides in the nucleus. Illumination with blue light triggers a conformational change in the LCB complex to expose the NLS motif, resulting in its nuclear translocation and the subsequent interaction between LexA and Lex binding sequence. Blue light can also induce the dimerization between CRY2 and CIB1, allowing the recruitment of VPR (a transcriptional activator of VP64-p65-RTA) to the transcription site of CAR. Therefore, the LINTAD system demonstrated that the CAR expression can be controlled by light with spatiotemporal resolutions.

In addition, an optogenetic strategy was developed to directly control the CAR functions by light. O’Donoghue et al. designed a split CAR system in which the Ag-recognition part of CAR was fused with iLID, while the other part containing signaling components was fused with iLID binding partner, SspB (O'Donoghue et al., 2021). This split CAR system can be functional when these parts are combined upon the illumination. Similarly, optoCAR was constructed from the split CAR system by substituting LOV2 in the iLID with circularly permuted LOV2 (cpLOV2) and incorporating a co-stimulatory domain 4-1BB into both segments of the split CAR (Figure 5B) (He et al., 2021). Another split design, light-switchable CAR (LiCAR), was created based on the LOV2-based iLID dimerization system. In this design, 4-1BB and CD3ζ are combined to be functional upon light stimulation (Nguyen et al., 2021). While these optogenetic strategies enabled precise spatiotemporal control of CAR-T functions *in vitro*, the limited ability of blue light to penetrate deep tissues may restrict their applications *in vivo*. To address this challenge, upconversion nanoparticles (UCNPs) were integrated into the LiCAR system to convert near-infrared (NIR) excitation into visible light emission (He et al., 2021; Nguyen et al., 2021). Nevertheless, these strategies have provided valuable insights into the design of light-inducible CARs to overcome the issues of on-target, off-tumor toxicity and have also demonstrated the potential for the application of other optogenetic tools.

## 7 Conclusion

Over the last few decades, a variety of genetically encoded biosensors and optogenetic tools have been developed to investigate complex molecular dynamics in T cell signaling pathways. In this review, we briefly overviewed the TCR/CAR signaling pathways (Section 2 and Section 3) and conventional methods to study these signaling pathways (Section 4). We then introduced general principles and examples of fluorescent biosensors (Section 5) and optogenetic tools (Section 6) in the research field of TCR/CAR signaling pathways.

Many biosensors designed for studying the TCR signaling pathway rely on FRET, utilizing two distinct FPs. However, this approach occupies a significant portion of the spectral space, limiting its application for multiplexed imaging. To overcome this, alternative single-color strategies, such as cpFP, dimerization dependent-dependent fluorescent protein (ddFP) (Alford et al., 2012) and homo-FRET can be employed for simultaneous visualization of multiple signaling activities within individual cells. Multiple fluorescent reporters has enabled multiplexed imaging involving up to six parameters within the same living cell (Mehta et al., 2018). Therefore, the development of single-fluorophore-based biosensors capable of simultaneous monitoring multiple signaling events will enhance our understanding of the relationships between diverse TCR/CAR signaling pathways with spatiotemporal resolutions.

While fluorescent biosensors offer valuable insights into local molecular dynamics, conventional light microscopy faces limitations in precision due to the diffraction limit (200–250 nm). Super-resolution imaging methods have significantly improved spatial resolution (10–100 nm) (Galbraith and Galbraith, 2011), providing valuable biological insights into TCR signaling pathways (Ritter et al., 2015; Barr et al., 2017; Wen et al., 2020). These imaging techniques, when combined with genetically encoded biosensors, can not only capture precise spatial information but also provide detail molecular dynamics such as protein-protein interactions or post-translational modifications in living cells. For example, Fluorescence Fluctuation Increase by Contact (FLINC)-based biosensors utilize fluctuations in fluorescence as a readout, enabling nanometer-sensitive measurement of the molecular activity through super-resolution microscopy (Mo et al., 2017; Lin et al., 2021).

In the field of CAR-T engineering, correct understanding of complex CAR signaling pathways and the optimal selection of the CAR domains is important to enhance the efficacy of CAR-T therapy. The fluorescent biosensors discussed in this review, originally designed for studying TCR signaling, can be further extended to investigate CAR signaling pathways. For instance, current CAR engineering methods have typically relied on *in vitro* assays or the expression of reporter genes or proteins such as IL-2, NFAT, or CD69. However, genetically encoded biosensors capable of measuring CAR activation and signaling upon antigen binding would enable the imaging-based selection of the most effective CAR construct in live cells. This offers a distinct advantage over traditional methods as it allows the simultaneous acquisition of sensitive information such as localization and activation kinetics.

Optogenetics provides a significant advantage by allowing precise control of specific protein activity, independent of other signaling pathways, with high spatiotemporal resoluations. Thus, optogenetic tools would be useful to examine the signaling pathways of TCR and CAR. In addition to their application in studying signaling pathways, optogenetic tools are widely used to mitigate side effects and enhance the effectiveness of CAR-T cell therapy. Light-responsive CAR-T cells, activated with blue light, allowed precise spatiotemporal functions of CAR at tumor sites (Huang et al., 2020; He et al., 2021; Nguyen et al., 2021; O'Donoghue et al., 2021). However, the delivery of blue light remains challenging, due to its inability to penetrate deep tissues. Therefore, it is also important to develop safe and efficient methods for light stimulation, such as UCNP, in order to achieve therapeutic effects. On the other hand, alternative strategies are emerging for *in vivo* applications in the immune system (Miller et al., 2021; Wu et al., 2021). Among these, focused ultrasound has significant potential for clinical applications, as it can penetrate any depth inside the human body using ultrasound devices. A recent study demonstrated the selective activation of CAR-T cells through focused ultrasound within specific tumor sites, resulting in a substantial decrease in on-target, off-tumor toxicity compared to conventional CAR-T cells (Wu et al., 2021).

In summary, genetically encoded biosensors and optogenetic tools are powerful tools that promise to deepen our understanding of signaling mechanisms in T cell and CAR-T cells. We anticipate that these tools will facilitate more sophisticated and comprehensive research, encompassing receptor triggering models and downstream signaling cascades of both T cells and CAR-T cells.

## Figures and Tables

**Table:** 

**AP-1**	activator protein 1
**APC**	antigen-presenting cell
**bNLS**	bipartite nuclear localization signal
**BRS**	basic residue-rich sequence
**CaM**	Ca^2+^ binding protein calmodulin
**CAR**	chimeric antigen receptor
**CD**	cluster of differentiation
**CFP**	cyan fluorescent protein
**CIB1**	cryptochrome-interacting basic-helix-loop-helix
**cpFP**	circular permuted fluorescent protein
**cSMAC**	central supramolecular activation cluster
**CRAC channel**	Ca^2+^ release-activated Ca^2+^ channel
**CRY2**	cryptochrome 2
**CryoEM**	cryogenic electron microscopy
**DAG**	diacylglycerol
**ddFP**	dimerization dependent-dependent fluorescent protein
**dSMAC**	distal supramolecular activation cluster
**ER**	endoplasmic reticulum
**ERK**	extracellular signal-regulated kinase
**FLIM**	fluorescence lifetime imaging microscopy
**FLINC**	fluorescence fluctuation increase by contact
**FP**	fluorescent protein
**FRET**	fluorescence resonance energy transfer
**GEF**	guanine-nucleotide-exchange factor
**Grb2**	growth factor receptor-bound protein 2
**hOPN4**	human melanopsin
**IP**_**3**_	inositol 1,4,5-triphosphate
**IP**_**3**_ **R**	inositol 1,4,5-triphosphate receptor
**IRAP**	insulin responsive aminopeptidase
**ITAM**	immunoreceptor tyrosine-based activation motif
**Itk**	IL2 Inducible T Cell Kinase
**KD**	dissociation constant
**LAT**	linker for the activation of T cells
**Lck**	lymphocyte-specific protein tyrosine kinase
**LOV**	light-oxygen-voltage-sensing
**M13**	myosin light-chain kinase
**MAPK**	mitogen-activated protein kinase
**MHC**	major histocompatibility complex
**mTFP**	monomeric teal fluorescent protein
**Nck**	non-catalytic region of tyrosine kinase
**NES**	nuclear export signal
**NF-kB**	nuclear factor kappa-light-chain-enhancer of activated B cells
**NFAT**	nuclear factor of activated T cells
**NK cell**	natural killer cell
**NMR**	nuclear magnetic resonance
**PHR**	photosensitive photolyase homology region
**PIP**_**2**_	phosphatidylinositol 4,5 biphosphate
**PKC**	protein kinase C
**PLCγ1**	phospholipase C gamma1
**pMHC**	peptide-MHC
**pSMAC**	peripheral supramolecular activation cluster
**R18**	octadecyl rhodamine B chloride
**RasGRP1**	Ras Guanyl Releasing Protein 1
**RK**	receptor kinase
**scFv**	single-chain variable fragment
**SH**	Src Homology
**SHP1**	Src homology region 2 domain-containing phosphatase-1
**SLP76**	cytoplasmic SH2 domain–containing leukocyte protein of 76 kDa
**SMAC**	supramolecular activation cluster
**smFRET**	single-molecule FRET
**Sos**	son of sevenless
**SPR**	surface plasmon resonance
**STIM1**	stromal interaction molecule 1
**TCR**	T cell receptor
**THEMIS**	thymocyte selection associated
**TRPC**	transient receptor potential channels
**UCNP**	upconversion nanoparticle
**UD**	unique domain
**YFP**	yellow fluorescent protein
**ZAP70**	zeta-chain-associated protein kinase 70
